# β-Tricalcium Phosphate as Alveolar Bone Grafting in Cleft Lip/Palate: A Systematic Review

**DOI:** 10.3390/dj11100234

**Published:** 2023-10-07

**Authors:** Alexander Patera Nugraha, Hui Yang, Junduo Chen, Kunhua Yang, Ploypim Kraisintu, Kyaw Zaww, Aobo Ma, Ruixian Wang, Nada Emad Alshafei Mohamed Alhadi, Juan Ramón Vanegas Sáenz, Guang Hong

**Affiliations:** 1 Division for Globalization Initiative, Liaison Center for Innovative Dentistry, Graduate School of Dentistry, Tohoku University, Sendai 9830865, Japanhong.guang.d6@tohoku.ac.jp (G.H.); 2 Department of Orthodontics, Faculty of Dental Medicine, Universitas Airlangga, Surabaya 60132, Indonesia

**Keywords:** medicine, β-tricalcium phosphate, bone graft, CL/P, dental material, bioceramic

## Abstract

The aim of this systematic review is to describe and identify the prospects of β-Tricalcium Phosphate (β-TCP) as an alveolar bone grafting (ABG) material in cleft lip/palate (CL/P) or alveolar bone cleft defects. A systematic review protocol based on the Preferred Reporting Items for Systematic Reviews and Meta-Analyses 2020 (PRISMA 2020) was drafted. The literature search was conducted using MEDLINE/PubMed, Web of Science/ISI Web of Knowledge, Scopus, and the Cochrane Library, with English as the inclusion criterion and no publication year limits. The keywords yielded a total of 5824 publications. After removing duplicates and non-English articles, there were 3196 suitable articles available for evaluation. Subsequently, 1315 studies remained after reviewing titles and abstracts. Furthermore, 85 full articles were assessed for eligibility. After reading the complete texts of those papers, 20 were eventually selected that matched the inclusion requirements. Thirteen out of the twenty studies included in this systematic review were deemed to have a low risk of bias; one had a high risk of bias; and six had a moderate risk of bias due to not reporting randomization. β-TCP, when used as an ABG material, is biocompatible, visible, practical, offers a less invasive procedure, and does not interfere with orthodontic treatment. Synthetic β-TCP for ABG can be an alternative to autologous bone grafts under certain terms and conditions. The efficacy of β-TCP for ABG in CL/P or alveolar bone cleft defects can be enhanced through a tissue engineering approach that combines β-TCP with growth factors, mesenchymal stem cells, or other graft materials, along with modifications to β-TCP’s physical properties.

## 1. Introduction

Cleft lip/palate (CL/P) is a major congenital birth defect in the craniofacial structure caused by a defect in palatogenesis during the embryonic phase. CL/P etiology is impacted by genetic, environmental, and a mixture of both factors. CL/P is shown clinically as a cleft in the lip, alveolar bone, palate, and nasal septum. The patients have cosmetic and functional deficiencies [1]. CL/P is one of the most frequent orofacial congenital abnormalities worldwide [2]. The epidemiology of cleft lip, cleft palate, and cleft lip and palate (CL/P) has been recorded at roughly 1 in 700 births, but it has also been reported at 1 in 500 to 1 in 2500 births in other parts of the world [3,4]. The prevalence of CL/P was estimated to be 10.8 million people in 2017, with a disease burden of 652.084 disability-adjusted life years in low- and middle-income countries (94.1%) [5]. CL/P is highly common in the Asia area, particularly in Japan, China, and Indonesia, which are high-risk nations for CL/P [6,7,8].

Lifetime costs, loss of productivity, lack of self-confidence due to facial, aesthetic, or cosmetic aspects, increased utilization of mental health services, speech and hearing impairment, risk of infection, and increased morbidity and mortality at all stages of life are all negative impacts of CL/P on the individual and society. Furthermore, it may affect the oral health-related quality of life of CL/P patients [9]. In individuals with CL/P, dental malformations are more prevalent due to anatomical abnormalities in the alveolar process. Approximately 83.3% of the individuals with CL/P had at least one dental anomaly, with tooth agenesis being the most prevalent abnormality observed. Furthermore, the group with unilateral CL/P had the greatest number of dental abnormalities [10]. These anomalies can create serious issues that can be avoided or mitigated through early detection and treatment.

The alveolar cleft volume increased with age in CL/P patients, which is related to an increasing breadth of the lip–palatal defect. Patients under the age of 18 had significantly higher rates of ipsilateral maxillary sinusitis, which may increase the risk of bone infection [11]. The alveolar process has an important role as the dentition host. Therefore, alveolar cleft that occurred in the CL/P needs to be closed. Alveolar bone grafting (ABG) and periosteoplasty are the two most frequent surgical methods that have been established for CL/P treatment. Regeneration of the alveolar cleft and continuous alveolar process so that teeth can erupt and be moved by means of orthodontics are the main objectives of ABG or periosteoplasty in CL/P patients. Secondary alveolar bone grafting (SABG) reconstructs the alveolus, supports permanent teeth, closes any residual anterior palatal fistulas, and supports the alar base and lip on the cleft side. Restoring maxillary integrity is also advantageous if future orthognathic surgery is necessary. The optimal period for alveolar bone grafting (ABG), according to the European and North American Cleft Association, is before canine eruption. However, various concerns remain, including the nature of the surgical and orthodontic techniques, the kind of bone and donor location, and the optimum approach to managing the space in the dental arch. Although the most typical age range for performing a bone graft is between the ages of 8 and 11, some hospitals have started to perform alveolar bone grafts at a younger age in the expectation of achieving better outcomes for unerupted incisors. A variety of donor sites have been used, but the iliac crest remains the most preferred, although it may pose challenges for some patients with medical conditions. Prior to ABG, several teams used orthodontics to rectify significant segment displacement or to align incisors to facilitate surgical access. Following ABG, lateral incisor absence can be addressed via orthodontic space closure, implant implantation, or bridgework [12].

The ilium is the most typical location for autologous ABG harvesting. Curettage, trapdoor or splitting procedures for cancellous bone, and the subcrestal-window approach for bicortical transplant are all ways of harvesting iliac ABG. However, potential consequences of using the ilium as a donor site may include discomfort, neurovascular damage, avulsion fractures of the ASIS, hematoma, infection, herniation of abdominal contents, gait impairment, cosmetic deformity, sacroiliac joint violation, and ureteral injury [13]. There is an option to replace the autologous iliac crest for ABG with a xenograft or synthetic graft.

**Figure 1:**
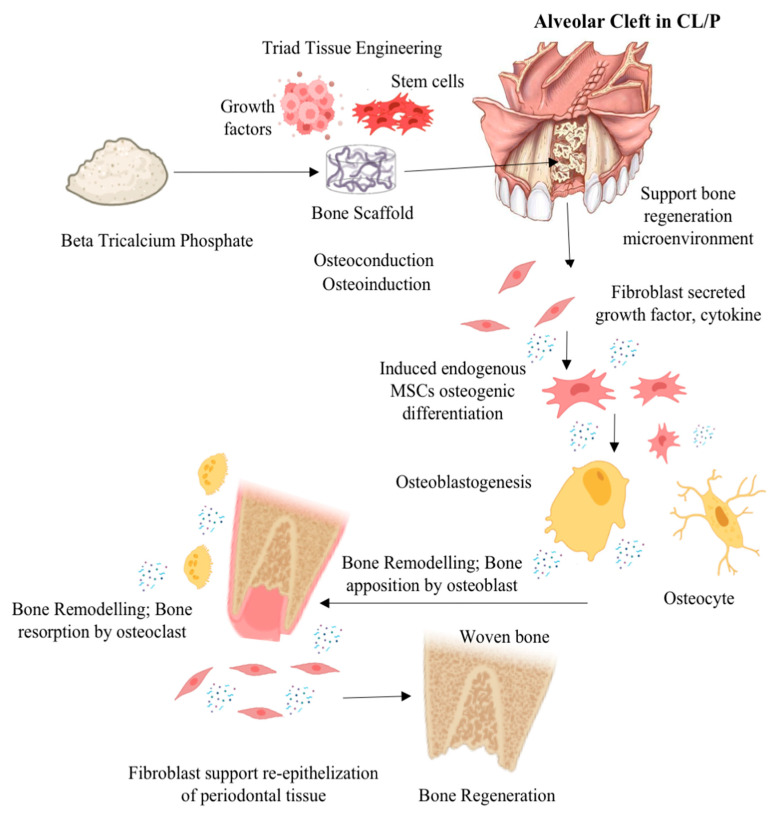
Illustration mechanism of action possibility β-TCP was used in a tissue engineering approach as an alveolar bone transplant in CL/P or an alveolar bone cleft defect (illustration image created with BioRender: Scientific Image and Illustration Software (https://www.biorender.com accessed on 30 June 2023) and alveolar bone cleft defect image from Texas Children’s Hospital (www.texaschildrens.org accessed on 30 June 2023).

Rapid integration of ABG is crucial for achieving structural stiffness. Structural and nonstructural ABG procedures modify alignment, function, and appearance by adding length, height, and volume. Corticocancellous autografts, allografts, xenografts, and synthetic grafts are all kinds of ABG. Autogenic grafts, which are harvested from the patient, are less likely to be rejected. However, the harvesting process adds an additional step, and donor site morbidity is prevalent. Secondary operations and donor site problems are avoided with allografts, xenografts, and synthetic grafts [14]. Stringent regulations are projected to significantly limit the allograft industry in the future. The use of xenograft or synthetic ABG, such as Beta (β)-tricalcium phosphate (β-TCP), a bioceramic biomaterial, is expected to be promising and helpful for clinical results in CL/P therapy. β-TCP materials, followed by xenograft biomaterials, which regrettably still lack established predictability and clinical efficacy, dominate the cranio-maxillofacial market [15]. Figure 1 illustrates the possible mechanism of action when β-TCP is used in a tissue engineering approach as an alveolar bone graft in CL/P or an alveolar bone cleft defect. Despite numerous efforts made to investigate the ABG in the field of cranio-maxillofacial medicine, the regenerative prospect of β-TCP as ABG in CL/P has not yet been fully elucidated and remains limited. Therefore, the aim of this systematic review is to describe the regenerative prospect of β-TCP as an ABG material in CL/P based on the existing literature.

## 2. Material and Methods

### 2.1. Focused Question

Following the Participants, Intervention, Control, and Outcomes (PICO) principle, a focused question was formulated before conducting the literature search according to Preferred Reporting Items for Systematic Reviews and Meta Analyses 2020 (PRISMA 2020). The focused question was ‘What is the prospect β-TCP as an ABG material to stimulate the regeneration of CL/P or alveolar bone cleft defects?’.

### 2.2. Search Strategies

A systematic review protocol based on PRISMA 2020 was drafted. In addition, reporting was based on the PRISMA 2020 checklist [16,17]. In addition, the systematic review was recorded in the international platform for registering systematic reviews and meta-analysis protocols (INPLASY) with registration number INPLASY202380113.

The following databases were searched: MEDLINE/PubMed (https://pubmed.ncbi.nlm.nih.gov accessed on 1 June 2023), Web of Science/ISI-Web of Knowledge (https://www.webofscience.com/accessed on 1 June 2023), Scopus (https://www.scopus.com/accessed on 1 June 2023), and the Cochrane Library (https://www.cochranelibrary.com/advanced-search accessed on 1 June 2023). Manual searches were undertaken to supplement the completed searches. Furthermore, the gray literature in The New York Academy of Medicine Gray Literature Report (http://www.greylit.org accessed on 1 June 2023) and the European System for Information on Gray Literature (http://www.opengrey.eu accessed on 1 June 2023) was screened [18]. Table 1 shows the search strategies in the selected databases.

### 2.3. Eligibility Criteria

The following categories of articles were included in this review: original articles that focused on the methodology of using β-TCP as an ABG material in animal models or humans to regenerate CL/P or alveolar bone cleft defects. Open access (accessed through the Graduate School of Dentistry, Tohoku University’s IP address) of full-text articles relevant to β-TCP ABG for CL/P or alveolar bone cleft defect were used as inclusion criteria. Reviews, short communications, editorial notes, processes, and recommendations were not considered and excluded. All types of experimental and observational studies in English were included. Nevertheless, no duplicate studies were included in the analysis. Adults or children of any gender or age are acceptable study subjects, as are any other objects of in vivo research. CL/P, alveolar cleft defect, β-TCP, and ABG, as well as any additional therapies involving tissue engineering, were included in the research as study factors or exposures. Bone regeneration, bone repair, bone volume, dentistry, bone remodeling, and any other measure of bone regeneration in CL/P were among the outcomes of the research examined. Articles in languages other than English, letters to the editor, and all types of reviews and commentaries were excluded. There were no restrictions on the year of publication, but only full papers could be accessed for free. The most recent search was conducted in June 2023.

### 2.4. Study Selection and Data Extraction

The three reviewers (A.P.N., H.Y., and J.C.) independently conducted electronic literature searches and selected the studies. Any disagreements were resolved by discussion or by consulting a second reviewer (J.R.V.S., G.H.) [16,17]. The reviewers (A.P.N., H.Y., and J.C.) worked to duplicate screening, extract, and recapitulate data using a standardized form in Microsoft Excel that had been validated prior to use [18]. Data was primarily extracted using the PICO protocol (Participants: patients (for clinical studies) or animals (for in vivo studies); Intervention: β-TCP ABG; Controls: autograft, xenograft, no treatment, or other regenerative materials; Outcomes: bone regeneration or bone remodeling of CL/P or alveolar bone cleft defects; Data relevant to methodology, sample size, duration of the studies, and the investigations carried out were extracted from each study. Results from the animal (in vivo) and human clinical studies were tabulated in the table using predetermined data collection forms by the two investigators independently [19].

### 2.5. Quality Assessment of Studies

Depending on the type, each study was assessed individually and independently by investigators. It was decided that for the quality assessment of any randomized clinical trials, the Consolidated Standards of Reporting Trials (CONSORT) would be used. The Animal Research: Reporting of In Vivo Experiments (ARRIVE) guidelines were selected for animal studies. Any disagreements were solved by discussion between investigators.

### 2.6. Risk-of-Bias Assessment

The risk of bias evaluation was carried out in accordance with a technique derived from prior systematic reviews [16,17,20]. This assessment evaluated the description of several quality assessment parameters, including a well-defined β-TCP as an ABG process, standardized sample or subject preparation, randomization of samples or subjects, tests conducted by a single blinded operator, a clear test method specification, and comprehensive reporting of results. The article was labeled “Y” for a given parameter if the authors reported it and “N” if the information could not be located. The articles were classified as having a high, medium, or low risk of bias based on the number of “Y” elements included (1–2, 3–4, or 5–6).

### 2.7. Statistical Methods

Microsoft Office Excel (2010, Microsoft, Chicago, IL, USA) was used for descriptive statistics. Due to the heterogeneity of the papers, a pairwise meta-analysis could not be performed.

## 3. Results

### 3.1. Study Selection, Data Extraction, and Quality Assessment

**Figure 2:**
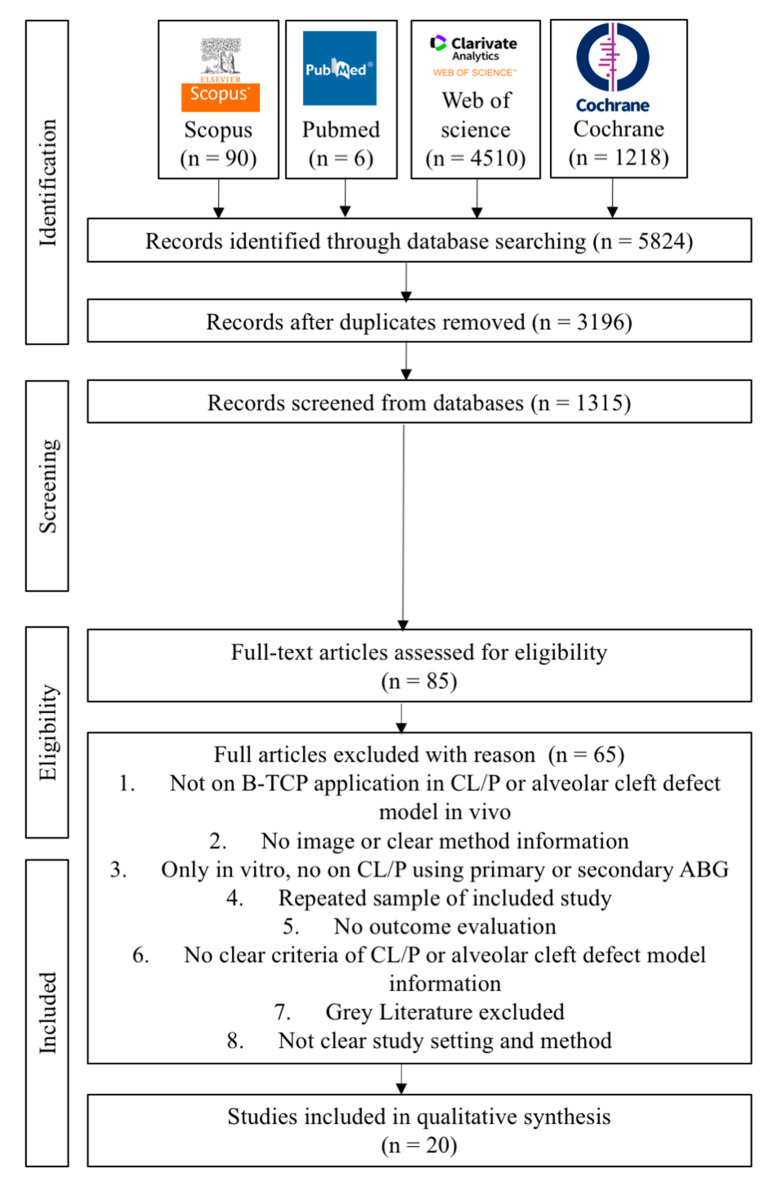
Flow diagram of PRISMA 2020 in the study selection process.

The keywords yielded a total of 5824 articles published, with 90 papers from Scopus, 6 papers from PubMed, 4510 papers from the Web of Science, and 1218 papers from the Cochrane Library, respectively. The 3196 suitable articles to evaluate after removing duplicates and languages We had 1315 studies left after doing title and abstract reading. Eighty-five full articles were assessed for eligibility. They read the complete texts of those papers and eventually chose 20 that matched the inclusion requirements. The reviewers (A.P.N., H.Y., and J.C.) independently performed critical evaluations utilizing JBI critical evaluation tools. Figure 2 depicts the flow diagram of the study selection process. A summary of descriptive characteristics of the articles included in this study is shown in Table 2 about animal model experiment. In addition, summary of descriptive characteristics of articles included in human clinical studies shown in Table 3.

### 3.2. Assessment of the Risk of Bias and Quality

The reviewers read the complete texts of those papers and eventually selected 20 studies that met the inclusion criteria. Thirteen of the twenty studies included in this systematic review had a low risk of bias, one had a high risk of bias, and six had moderate bias. Most of the studies did not report the randomization, which is considered a potential source of bias (Table 4).

### 3.3. Qualitative Analysis

Most of the studies included in this systematic review were experimental studies using animal models of alveolar bone cleft defects that were representative of CL/P patients. There were fourteen studies using an alveolar bone cleft defect animal model. Six studies used dogs [24,25,26,29,31,34], five studies used rats [21,22,27,28,33], two studies used goats [30,32], and only one study used rabbits [23]. Only one research study claimed that the rat’s palatine fissure was an acceptable location for the bone substitute implant application of bone graft materials to replicate the human alveolar cleft in animal model studies of CL/P. In addition, the palatine fissure is a congenital bone abnormality comparable to the alveolar cleft abnormalities seen in people [27]. Nevertheless, the rest of the studies were successfully established and reported the research outcome for the alveolar bone cleft defect animal models. A preliminary or pre-clinical study using an animal model as a representative of CL/P conditions in patients is important before the clinical application of the proposed ABG in the clinical setting.

On the other hand, there were several studies of β-TCP application in CL/P patients in a clinical setting, such as five articles of case–control clinical studies [25,32,37,38,40] and one article of a retrospective cohort study [35]. Most clinical setting studies of CL/P focused on unilateral alveolar cleft [25,36,37,38,40]. However, only one study used the unilateral or bilateral cleft patients as the study participant in the retrospective study setting [35]. The most frequent prevalence of CL/P type in patients was unilateral alveolar cleft. Out of the 20 articles included in this systematic review, 6 articles also investigated the potential impact of alveolar bone graft (ABG) application on orthodontic treatment [21,28,29,30,31,36,40]. β-TCP is a more biocompatible alternative to autogenous bone transplantation for orthodontic tooth movement in alveolar bone cleft abnormalities [29]. Other earlier research has found that the use of β-TCP does not impair orthodontic treatment [30,31,40].

In terms of bone regeneration of CL/P or alveolar bone defect clefts, autologous bone grafts are considered the gold standard, as indicated by several studies [22,27,28,33]. Most of the studies used the autologous bone grafts from hip bones [21], iliac crest bones [26,30,31,32,34,35,37], tibia bones [24,39], particulate marrow and cancellous bone [29], and mandibular symphysis bone [38,40]. However, two articles do not use any gold standard ABG in animal models or as control groups [23,36]. Autograft and xenograft seemed to be interchangeable terms; after more tissue engineering adjustments, synthetic β-TCP and HA might be used as an alternative ABG [21].

Some studies suggested β-TCP, which is a bioceramic, as an ABG material for bone regeneration in CL/P or alveolar bone cleft defects. Notwithstanding this, β-TCP alone cannot substitute for autologous ABG [21]. The result of a previous study found a lower bone volume (BV) and bone mineral density (BMD) after β-TCP application compared to autologous ABG [27]. β-TCP may be able to overcome its lack of osteoinductive and osteoconductive qualities after undergoing many physical changes or the inclusion of certain stem cells or growth factors. There are several modifications of β-TCP as the alternative ABG, such as the combination of rhBMP2 in a β-TCP scaffold [35], AMSCs seeded in β-TCP [22,24], BMSCs or BMMNSCs combined with β-TCP granules [26,31,34,37], β-TCP in microporous [25,32,36], composite xenogeneic dentin with β-TCP [23], the mixture of autologous ABG in β-TCP granules [32,38,40], combination of HA-β-TCP [28], col/β-TCP scaffolds, and PLLA/PCL scaffolds [33]. Even though autografts are the best option for bone regeneration, tissue-engineered β-TCP might be a viable alternative, particularly if autografts are hard to come by or there is donor site morbidity [39]. Thus, it may decrease the surgical pain and the number of hospital stay days [37].

Various examination methods are proposed to investigate the efficacy of β-TCP as ABG in the clinical setting or pre-clinical research, such as µCT, histology analysis, CBCT, RT-qPCR, occlusal digital radiography, and densitometry with computer-aided software. µCT was used to examine BMD, BV/TV, BF, defect size, Hounsfield unit, bone quality, trabecular thickness of bone, mineralized matrix formation, and bone mineral content [21,22,23,27,28,32,33,34]. Several important osteogenic biomolecular markers, such as Runx2, OSC, SPARC, BSP, ALP, and Osx mRNA, can be analyzed by RT-qPCR [33]. Histopathology analysis was conducted to investigate several biomarkers within the tissue, such as lamellar bone and woven bone21, runx2, ALP, Osx, BMP2, TRAP expression, osteoblast and osteoclast number, tooth movement, and root resorption, bone formation (%), residual graft (%), collagen regeneration (%), and defect area healing (%) [22,23,24,27,28,30,31,33,34,39]. The CBCT instrument can be used to investigate preoperative defect volume, postoperative residual defect volume, bone formation, residual calcified tissue, spontaneous eruption of the canine or lateral incisor, continuous alveolar process, residual oronasal fistula, BV/TV, BFR, bone union, Chelsea scale Tb. Th, Tb.N, Tb.Sp, TBPf, SMI, FD, cleft volume pre-operation, and graft volume post-operation [21,23,25,36]. Conventional dental occlusal radiographs have been used to analyze the height of the bone graft, alveolar height and eruption disturbance, bone deposition, repaired alveolar cleft, and residual alveolar height (%) [26,29,31,40]. In addition, bone density examination can be investigated by means of densitometer software with digital radiography [24]. Direct observation, such as duration of hospital stay, may be useful to know the efficacy of ABG administration in CL/P [23]. Those various examination methods help to elucidate the β-TCP as ABG in the CL/P patients or alveolar bone cleft defect in vivo.

## 4. Discussion

The objective of alveolar cleft repair is to reconstruct bone volume and quality to match the original anatomy. The accompanying soft-tissue attachment of the oral and nasal mucosa is specific to these cleft abnormalities [21]. This systematic review aimed to provide a summary of existing research on the regeneration possibility of β-TCP as ABG in cleft lip and palate patients or alveolar bone cleft defect animal models, assessing the success or failure of these interventions. Overall, the findings of this comprehensive analysis show that the use of β-TCP as ABG improves the regeneration of alveolar bone cleft defects in CL/P humans or animal models (in vivo) [21,22,23,24,25,26,27,28,29,30,31,32,33,34,35,36,37,38,39,40].

Nevertheless, a downside of β-TCP alone cannot substitute the golden standard of ABG, which is autologous bone graft [27]. Autologous bone remains the gold standard for cleft repair. However, autografts have certain drawbacks, including restricted bone supply, the requirement for an extra donor site, the accompanying postoperative morbidity (pain, hematoma, and delayed ambulation), and an intrinsic sensitivity to resorption in the long run [21]. Therefore, different tissue-engineered bone replacements have been proven to be effective options for encouraging bone fusion and minimizing donor site morbidity. The β-TCP is a bioceramic, a synthetic ABG described, and has been recognized to be an alternative to autologous or xenograft ABG, which is acceptable in numerous terms and situations [25]. β-TCP with tissue engineering modification eliminates the limits of autologous bone grafts, such as limited donor supply and donor site morbidity, and minimizes the patient’s surgical stress, which may be connected to a decreased operating time and hospital stay duration reported [23].

Alternatively, establishing appropriate β-TCP resorption characteristics, the optimal balanced ratio of HA and β-TCP varies between 65:35 and 55:45 [41,42]. Some MSCs, such as AMSCs or BMSCs, may boost the bone regeneration ability of β-TCP [22,24,26,31,34,37].

In addition, the alteration of β-TCP’s shape or combination with growth factor, substance, or collagen is also promising for enhancing the osteoinductive and osteoconductive potential of β-TCP [23,25,32,33,35,36,38,39,40]. To date, research has not been carried out to examine the ideal qualities of β-TCP. Additionally, there is limited research that has investigated the clinical effectiveness of β-TCP in human subjects in a clinical setting [35,36,37,38,39,40]. Animal research included in this comprehensive review demonstrates that β-TCP stimulates bone regeneration in CL/P or alveolar bone cleft defects [21,22,23,24,25,26,27,28,29,30,31,32,33,34]. In addition, most of these studies examined the efficacy of the β-TCP and presently utilized therapies such as hip bone, iliac crest bone, tibia bone, particulate marrow and cancellous bone, and mandibular symphysis bone (all of which have been used clinically) [21,24,26,30,31,32,33,34,35,37,38,39,40]. Nevertheless, this present study discovered in those two articles that there was no gold standard for ABG usage within the research [23,36]. Therefore, future animal studies should evaluate the in vivo effectiveness of β-TCP with the aforementioned materials and methodologies before being approved in clinics.

Regarding the post-operative or post-treatment examination of newly produced bone or bone regeneration, the majority of the included research employed CBCT for clinical investigations and micro-CT or HPA for in vivo studies. We found that five studies used CBCT to examine bone regeneration in post-operative CL/P patients, and four studies used conventional dental occlusal radiographs [26,29,31,40]. CBCT scans provide a reduced radiation dosage and a limited scanning time (10–70 s), and they allow doctors to scan a small region for a specific diagnosis with fewer picture artifacts [43,44,45]. Orofacial cleft patients require a 3D study for the right diagnosis as they present with numerous medical disorders, including bone graft operations, impacted teeth, or supernumerary teeth. This is the reason why CBCT is advised for orofacial cleft patients by the European Academy of Dental and Maxillofacial Radiology [46]. Therefore, additional research should employ CBCT as an evaluation technique to quantify newly produced bone, especially in clinical situations. Thus, the registration of the protocol is encouraged and will strengthen its robustness in subsequent studies. Although systematic reviews are regarded as the most solid evidence, the research included in each systematic review also has a related bias. The methodological variability includes discrepancies in the trial locations, a lack of a priori acceptable sample size estimates, the kind of sample included (e.g., type of cleft, age groups), intervention protocols, bone measuring techniques, and follow-up timeframes. Other factors may alter the analysis of primary outcomes as they affect bone remodeling, notably the location of teeth on the bone graft, the cleft defect’s breadth, and the volume of grafted bone [47].

The most apparent source of autologous ABG would be the iliac crest bone, which may be retrieved by a surgical procedure with a risk of morbidity, such as infection. However, employing this option in every circumstance would not be practicable, and a synthetic ABG such β-TCP would be more sensible [21,26,30,39]. In the research evaluated, there were various constraints that may have biased outcomes. With the information currently available from in vivo and clinical studies, the overall effect summary of β-TCP as ABG for CL/P or alveolar bone cleft defect cannot be determined. Furthermore, these findings should be regarded with caution since clinical and in vivo methodological heterogeneity might alter the extent of the statistical heterogeneity revealed. This systematic review found multiple heterogeneity variables, such as the number of participants or samples, kind of cleft defect, different treatment, timing of outcome, and intervention design. In the present review, we noticed that six of the studies did not apply any type of randomization [24,35,36,38,39,40]. A lack of randomization may have altered the direction of results due to examiners’ bias.

The primary results may also be altered by the clinician’s skill and the study group’s scientific competency. Secondarily, most selected studies were classified as having poor or middling overall quality, which may lower confidence in the findings [47]. Moreover, the included studies might overstate the impact of the findings due to the inclusion of numerous publications from a single research project or by ignoring studies in other languages. In this systematic review, the majority of the studies employed a small sample size. In addition, varied and diverse criteria for CL/P patient selection could have had an impact on the estimated efficacy of β-TCP as an ABG material. Nevertheless, there is no gold standard for alveolar bone cleft defect size in animal models. Because of the variability in the techniques, measurements, and findings, it was not possible to perform a meta-analysis in this systematic review. A systematic review without meta-analysis may carry a substantial risk of bias. This was likely the most significant shortcoming of our review, as the mean overall effectiveness of β-TCP could not be calculated. Future studies should conduct blinded RCTs to control various sources of bias, such as the randomization technique, assessment equipment, and follow-up timeframes. Moreover, the cost–benefit analysis of these β-TCP to be employed in tissue engineering procedures for regeneration strategies in the ABG of CL/P is advocated as it plays a vital role in healthcare regulation.

## 5. Conclusions

The following findings may be drawn from this systematic review:

- β-TCP as an ABG material is biocompatible, more visible and practical, offers a less invasive procedure, and does not interfere with orthodontic treatment.
- β-TCP as a synthetic ABG material can be the alternative to autologous bone grafts with several terms and conditions, such as if autografts are hard to come by, there is donor site morbidity, and the size of the defect restricts the size of the autograft.
- The enhancement of osteoinductive and osteoconductive abilities for improvement of β-TCP efficacy for ABG in CL/P or alveolar bone cleft defects can be achieved via a tissue engineering approach combining β-TCP with growth factor, mesenchymal stem cells, or other graft materials and the modification of β-TCP physical properties.

However, due to several research gaps concerning the original studies’ methodological quality, heterogeneity and lack of findings, conclusions should be regarded with care.

## Figures and Tables

**Table 1:** Databases and search strategies of this study.

Database	Search Strategy
Scopus	β-tricalcium AND phosphate OR β-TCP or bone graft OR bone grafting OR alveolar AND bone AND graft AND alveolar AND bone AND cleft OR cleft AND palate AND (LIMIT-TO (PUBSTAGE, “final”)) AND (LIMIT-TO (OA, “all”)) AND (LIMIT-TO (DOCTYPE, “ar”)) AND (LIMIT-TO (LANGUAGE, “English”)) AND (LIMIT-TO (SRCTYPE, “j”))
PubMed	β-tricalcium phosphate OR β-TCP OR bone graft OR bone grafting OR biomaterial OR alveolar bone graft AND alveolar bone cleft AND cleft palate Filters applied: Free full text, Clinical Trial, Randomized Controlled Trial, English, Exclude preprints, MEDLINE.
Web of Science	((((((ALL = (β-tricalcium phosphate)) OR TS = (β-TCP)) OR TS = (bone graft)) OR TS = (bone grafting)) OR TS = (alveolar bone graft)) AND TS = (alveolar bone cleft)) OR TS = (cleft palate) Refined By: Open Access. Click to remove this refine from your search. Document Types: Article. Click to remove this refine from your search. Open Access: All Open Access. Click to remove this refine from your search. Languages: English.
Cochrane Library	β-tricalcium phosphate OR β-TCP OR bone graft OR bone grafting OR alveolar bone graft AND alveolar bone cleft OR cleft palate in Title Abstract Keyword—in Trials, Clinical Answers (Word variations have been searched)

**Table 2:** Summary of descriptive characteristics of articles included in animal model experiments.

Authors, Year, Country	Study Design	Sample/ Subject Criteria (n)	Type of Cleft	Type of Tissue Engineering	Examinations and Variables	Outcome	Conclusion
Möhlhenrich et al., 2021, German [21]	True experimental, post-test-only control group design	Twenty-one 8-week-old male Wistar-HAN rats; average weight of 465 ± 34 g.	1.7 mm alveolar clefts at the left side of the upper jaws with 0.14 N continuous orthodontic tooth movement application	Autologous rat hip, xenograft human bone substitute material and synthetic graft β-TCP and HA.	µCT: BMD, BV/TV. Histomorphometric analysis: lamellar bone and woven bone	Autologous ABG better than synthetic ABG in new bone formation, is less resorbed, and is better integrated into the cleft defect.	β-TCP and HA combined with growth factors and cells might be used as an alternative to autologous ABG.
Putri et al., 2022, Indonesia [22]	True experimental, post-test-only control group design	Thirty-six male Wistar rats (*Rattus novergicus*)	5 × 5 mm alveolar defect of the upper jaws	Autologous rat alveolar bone graft (ABG), human cancellous freeze-dried graft (HCG)–human adipose stem cell (hADSC), β-TCP–hADSC	Immunohistochemical analysis: runt-related transcription factor 2 (RUNX2), alkaline phosphatase (ALP), osterix (OSX), and bone morphogenetic protein 2 (BMP2) µCT: BV/TV (mm^3^), BF (%), and trabecular thickness of bone (TT, mm).	RUNX2, OSX, ALP, and BMP2 expression was enhanced in HCG-hADSC compared to β-TCP-hADSC and ABG.	Exogenous hADSC improved the ability of HCG and β-TCP to enhance osteogenesis, osteoconduction, and osteoinduction.
Kamal et al., 2017 German [23]	True experimental, post-test-only control group design	Sixteen male New Zealand rabbits.	Unilateral alveolar cleft defects	β-TCP, composite xenogenic dentin with β-TCP.	µCT: Defect size (mm^3^) Hounsfield unit (HU) % BV/TV, BMD. histomorphometric analysis: % bone formation % residual graft.	Dentin/β-TCP group showed significantly larger bone volume fraction (%) and residual graft (%) compared to β-TCP group.	Alveolar cleft defects repaired with dentin/β-TCP resulted in a larger graft residual volume and bone volume fraction.
Shahnaseri et al., 2020; Iran [24]	True experimental, post-test-only control group design	Four male canines	Unilateral alveolar bone cleft (15 mm)	Autologous AMSCs osteogenic differentiated seeded in HA/β-TCP scaffold, autologous tibia bone graft	Densitometer software with digital radiography: bone density histomorphometric: Bone regeneration (%)	Bone density and bone regeneration in autologous tibia bone grafts and autologous AMSCs with osteogenic differentiation planted in HA/β-TCP scaffolds did not differ in a statistically meaningful way.	Autologous AMSCs that are osteogenically differentiated and seeded in HA/β-TCP scaffolds can be used to reconstruct bone defects in patients who are unable to receive autogenous bone grafting when the size of the defect restricts the size of the autograft.
Pourebrahim et al., 2013; Iran [25]	True experimental, post-test-only control group design	Four male mongrel dogs	15 mm alveolar cleft in the crest to nasal floor via removal of two of the three incisors bilaterally	Autogenous tibial graft, HA/β-TCP loaded canine AMSCs	Histomorphometric: bone regeneration (%) and collagen regeneration (%)	At 15 and 60 days, the autograft sides had more bone growth than the stem cell sides, at 45% and 96%, compared to 5% and 70%, respectively, with significant differences between groups.	Tissue-engineered HA/β-TCP-loaded cAMSCs might be a viable alternative, particularly if autografts are hard to come by or there is donor site morbidity.
Huang et al., 2015; China [26]	True experimental, post-test-only control group design	Fourteen 24-week-old male beagles	Unilateral alveolar bone defect with 15 mm size	Autogenous iliac crest bone graft; tissue-engineered bone (TEB) BMSCs/β-TCP with rapid maxillary expansion (RME)	Occlusal radiograph: height of the bone graft; Histomorphometric: bone formation (%)	In comparison to untreated dogs or dogs just receiving autogenous iliac bone after 8 weeks of therapy with TEB BMSCs-β-TCP, and RME, the dogs’ new bone production and mineralization were dramatically accelerated.	BMSCs-β-TCP also have the capacity to replace autogenous bone, and their combination with RME may be another option for treating alveolar clefts.
Ito et al., 2019; Japan [27]	True experimental, post-test-only control group design	Twenty male Sprague Dawley rats	Alveolar bone cleft in the palatine	Autogenous bone graft, β-TCP.	µCT: BV and BMD Histology analysis: Osteoblast, osteoclast, alkaline phosphatase, tartate-resistant acid phosphatase	Autologous bone grafts had a considerably larger bone volume and BMD than β-TCP.	β-TCP resulted in lower bone volume and BMD than autologous bone transplants.
Möhlhenrich et al., 2022 [28]	True experimental, post-test-only control group design	Twenty-one male Wistar rats (*R. novergius*)	Alveolar bone cleft	Autografts, human xenografts and synthetic bone substitute β-TCP/HA	μCT and histopathological investigation: tooth movement, and root resorption.	The differences in root resorption and tooth movement between the bone graft replacements and autologous bone were not statistically significant at any time.	Autografts, human xenografts, and synthetic bone substitutes used for cleft repair all appear to have a comparable influence on later orthodontic tooth movement and root resorption.
Hossain et al., 1996; Bangladesh [29]	True experimental, post-test-only control group design	Nine male beagles dog	Alveolar bone cleft	Autogenous particulate marrow and cancellous bone (PMCB), β-TCP and combination with experimental tooth movement	Radiograph analysis: bone deposition Histopathological investigation: bone regeneration	β-TCP showed a more pronounced biodegradative reaction to orthodontic force in connection with the production of new cementum. Root resorption was considerably lower in the β-TCP region than in the PMCB zone.	β-TCP is a more biocompatible option for autogenous bone transplantation into alveolar bone cleft defects that support orthodontic tooth movement.
de Ruiter et al., 2011; Netherland [30]	True experimental, post-test-only control group design	Ten adult Dutch milk goats (*Capra hircus*)	Alveolar bone cleft	β-TCP, autologous iliac crest bone graft	Histologic assessment: new bone formation and bone graft resorption. Radiographic measurement: orthodontic tooth movement.	An average tooth movement of 43.2% was measured in clefts restored with iliac bone and 41% in clefts rebuilt with β-TCP.	The bone substitute β-TCP is at least as successful as autologous iliac crest bone in the healing of alveolar clefts in goats, according to surgical, orthodontic, histologic, and radiologic viewpoints.
Zhang et al., 2011; China [31]	True experimental, post-test-only control group design	Six canines	Alveolar bone cleft	Porous β-TCP combined with osteogenically induced BMSCs and autologous iliac bone with experimental tooth movement	Occlusal radiographic: repaired alveolar cleft, residual alveolar height (%). Immunofluorescence: rate of bone formation and mineralization. Histological examination: area of the residual scaffold in the grafted region (%) and area of bone formation in the grafted region (%)	When compared to β-TCP alone, which was absorbed severely, BMSC-porous b-TCP significantly encouraged new bone formation and mineralization and attained a satisfactory height of the repaired alveolar cleft.	For patients with alveolar clefts and resultant orthodontic tooth movement, porous β-TCP in combination with osteogenically produced BMSCs may be a practical therapeutic technique as a replacement for bone transplants.
Janssen et al., 2017, Netherland [32]	True experimental, post-test-only control group design	Ten Dutch milk goats	Alveolar bone cleft defect	Microstructured beta-tricalcium phosphate (β-TCP) putty with autologous iliac bone	Histomorphometric and μCT: bone quality and BV/TV	There was no statistically significant difference between cleft sites and bone area percentages in β-TCP-CMCG and autologous bone grafts.	β-TCP-CMCG putty provides superior surgical handling in the correction of alveolar cleft deformities.
Ekin et al., 2015; Turkey [33]	True experimental, post-test-only control group design	Fifty-six Sprague Dawley rats	Critical-sized alveolar bone cleft defect	autograft, col/β-TCP scaffolds, and PLLA/PCL scaffolds	μCT: mineralized matrix formation, new bone formation, BV HPA: defect healing and new bone formation. RT-qPCR: Runx2, OSC, SPARC, BSP, ALP, and OSX	The autograft group had the greatest new bone volume rate at 1 month and 4 months.	The synthetic tissue scaffolds reported herein have significant potential as an alternative treatment option when cost, donor region morbidity, and hospitalization time are considered.
Tokugawa et al., 2012; Japan [34]	True experimental, post-test-only control group design	Ten female beagles dogs	Alveolar bone cleft defect	Canine BMSCs cultured on β-TCP, β-TCP.	μCT: BMD (mg/cm^3^) Bone mineral content (mg/mm); histopathological investigation: bone regeneration.	The regenerated bone in the MSCs/β-TCP group exhibited a bone mineral density that was midway between that of normal bone and that of β-TCP only.	cBMSCs-β-TCP-based bone regeneration offers a less invasive alternative to standard cancellous iliac bone autografts for alveolar bone replacement.

**Table 3:** Summary of descriptive characteristics of articles included in human clinical studies.

Authors, Year, Country	Study Design	Sample/ Subject Criteria (n)	Type of Cleft	Type of Tissue Engineering	Examinations and Variables	Outcome	Conclusion
Trujillo et al., 2018, USA [35]	Retrospective cohort study	Twenty-five patient CL/P: 15 and 10 females	Unilateral or bilateral clefts.	Iliac crest bone autograft Mandibular symphyseal bone graft, rhBMP-2/β-TCP bone substitute.	CBCT: Preoperative defect volume (cm^3^), postoperative residual defect volume (cm^3^), bone formation (%)	The bone formation was shown in the iliac crest group, which was followed by the rhBMP-2/ACS/β-TCP group and the mandibular symphysis group but was not significantly different between groups.	In alveolar cleft patients, rhBMP2 administered in a β-TCP scaffold may be an effective substitute for autogenous iliac crest.
Janssen et al., 2019, Netherland [36]	Case–control randomized clinical study,	Total of 20 CL/P patients: 14 males and 6 females	Unilateral CL/P	Microporous β-TCP	CBCT: Residual calcified tissue, spontaneous eruption of canine/lateral incisor, continuous alveolar process, residual oronasal fistula	No significant granule loss, surgical infection, or wound dehiscence occurred. The operating patients, who had an average residual calcified tissue volume of 65% one year after surgery, had no orinasal fistulas left.	In the clinical setting, SABG using microporous β-TCP is safe to utilize.
Du et al., 2017; China [37]	Clinical study	Ten CL/P patients (5 males and 5 females)	Unilateral alveolar cleft defects	Autologous iliac crest bone graft (ICBG), bone marrow mononuclear cells (BMMNCs) combined with β-TCP granules	CBCT and computer-aided engineering technology: bone volume (mm^3^), bone formation ratio (%), Bone volume (mm^3^) bone Formation ratio (%). bone union; Chelsea scale; duration of hospital stay (days).	Average defect volume, bone formation ratio (%), bone volume (mm^3^), and bone formation ratio (%) were not significantly different between groups.	Alveolar cleft repair with autologous BMMNCs and β-TCP granules was radiographically similar to using ICBG.
Miyagawa et al., 2020; Japan [38]	Clinical study	Thirty-one CL/P patients	Non-syndromic unilateral cleft lip and alveolus (UCLA) and cleft lip palate (UCLP)	Iliac crest bone, Mandibular symphysis, Mandibular symphysis combined with β-TCP granules.	CBCT: BV/TV, trabecular thickness (Tb.Th), trabecular number (Tb.N), trabecular separation (Tb.Sp), trabecular bone pattern factor (TBPf), structure model index (SMI), and fractal dimension (FD).	TBPs revealed variations between the IC and MS groups, leading to higher bone volume density values and a lower TBPf value in the IC group when compared to the MS group.	The use of β-TCP granules could produce similar results in the microstructure of the bone bridge.
de Ruiter et al., 2015; Netherland [39]	Prospective clinical study	Seven CL/P patients (5 males and 2 females)	Unilateral alveolar cleft	Micro-structured β-TCP	CBCT: cleft volume pre-operation; graft volume post-operation; bone volume 6 months post-operation	The bone volume thus gained was satisfactory six months following the surgical grafting of micro-structured β-TCP into the alveolar cleft: comparing the average bone volume to the initial cleft volume, 73% to 6%.	The therapeutic application of microstructured β-TCP bone replacement in alveolar cleft repair.
Weijs et al., 2010; Netherland [40]	Clinical study	Forty-seven CL/P patients (24 males and 23 females)	Unilateral alveolar cleft	Autogenous mandibular symphyseal bone only, mandibular symphyseal bone wrapped in /β-TCP granules	Occlusal radiograph: alveolar height and eruption disturbance.	There was no discernible difference in alveolar height or eruption disruption between the β-TCP granule group and the mandibular symphysis bone alone.	Autogenous mandibular symphyseal bone grafts enhanced with β-TCP granules can be utilized effectively in circumstances when the alveolar cleft is too big to be grafted with mandibular symphyseal bone alone.

**Table 4:** Individual studies are prone to bias. JBI critical appraisal for prevalence data studies.

Authors, Year, Country	CL/P or Alveolar Cleft Defect	β-TCP Utilization	Sample Preparation	Randomization	Blind Examiner	Test Method Clearly Reported	Complete Results	Risk of Bias
Möhlhenrich et al., 2021, German [21]	Y	Y	Y	Y	Y	Y	Y	Low
Putri et al., 2022, Indonesia [22]	Y	Y	Y	Y	Y	Y	Y	Low
Kamal et al., 2017 German [23]	Y	Y	Y	Y	Y	Y	Y	Low
Shahnaseri et al., 2020; Iran [24]	Y	Y	Y	N	N	N	N	High
Pourebrahim et al., 2013; Iran [25]	Y	Y	Y	Y	Y	Y	Y	Low
Huang et al., 2015; China [26]	Y	Y	Y	Y	Y	Y	Y	Low
Ito et al., 2019; Japan [27]	Y	Y	Y	Y	Y	Y	Y	Low
Möhlhenrich et al., 2022 [28]	Y	Y	Y	Y	Y	Y	Y	Low
Hossain et al., 1996; Bangladesh [29]	Y	Y	Y	Y	N	N	Y	Moderate
de Ruiter et al., 2011; Netherland [30]	Y	Y	Y	Y	Y	Y	Y	Low
Zhang et al., 2011; China [31]	Y	Y	Y	Y	Y	Y	Y	Low
Janssen et al., 2017, Netherland [32]	Y	Y	Y	Y	Y	Y	Y	Low
Ekin et al., 2015; Turkey [33]	Y	Y	Y	Y	Y	Y	Y	Low
Tokugawa et al., 2012; Japan [34]	Y	Y	Y	Y	Y	Y	Y	Low
Trujillo et al., 2018, USA [35]	Y	Y	Y	N	N	Y	Y	Moderate
Janssen et al., 2019, Netherland [36]	Y	Y	Y	N	N	Y	Y	Moderate
Du et al., 2017; China [37]	Y	Y	Y	Y	Y	Y	Y	Low
Miyagawa et al., 2020; Japan [38]	Y	Y	Y	N	N	Y	Y	Moderate
de Ruiter et al., 2015; Netherland [39]	Y	Y	Y	N	N	Y	Y	Moderate
Weijs et al., 2010; Netherland [40]	Y	Y	Y	N	N	N	Y	Moderate

## Data Availability

Data are available on request to the corresponding author by e-mail (alexander.patera.nugraha@fkg.unair.ac.id or hong.guang.d6@tohoku.ac.jp). Following PRISMA checklist for systematic review available at: http://prisma-statement.org/prismastatement/checklist.aspx? Accessed on 1 June 2023.
